# Supplementary material for: Real-time multispeckle spectral-temporal measurement unveils the complexity of spatiotemporal solitons
Source: Nat Commun. 2021 Jan 4;12:67. doi: 10.1038/s41467-020-20438-z (PMC7782776; doi:10.1038/s41467-020-20438-z)
Supplement: Supplementary file 1 — Supplementary Information [file 41467_2020_20438_MOESM1_ESM.pdf]

Supplementary Information

Real-time multispeckle spectral-temporal measurement unveils the complexity of spatiotemporal solitons

Guo, Wen and Lin et al.

Supplementary Note 1: Full configuration of the experimental system.

The schematic diagram of the experimental system is presented in **Supplementary Figure 1**. The system consists of a spatiotemporal mode-locking (STML) multimode fiber laser and the multispeckle spectral-temporal (MUST) measurement system. The multimode laser has a ring cavity. An Yb-doped gain fiber (Nufern LMA-YDF-15/130-VIII, 5 m length, 15 μm core size) is pumped by a multimode laser diode (~30 W maximum power, 976 nm wavelength) through a signal-pump combiner (SPC) with a multimode pigtail (Nufern LMA-GDF-15/130 0.08/0.46NA, ~2 m length, 15 μm core size). A multimode grade-index (GRIN) fiber (Thorlabs GIF625, 2.5 m length, 62.5 μm core size) is fusion-spliced to the gain fiber, where a large core offset is applied to excite the higher-order modes. The numbers of modes supported by the optical fibers used in the cavity are estimated and summarized in **Supplementary Table 1**. Two collimators (Cols) are utilized to launch the lightwave into free space and couple it back into the fiber. Two half-wave plates (λ/2) and two quarter-wave plates (λ/4) are used for polarization control, which is in conjunction with a narrow bandpass filter (F, Semrock LL01-1064-12.5, ~4 nm bandwidth) and a polarization-dependent isolator (ISO) for realizing the STML operation. A 50:50 beam splitter (BS) is employed to extract the laser signal. The roundtrip time of the laser cavity is about 47.8 ns, corresponding to a fundamental repetition rate of ~20.9 MHz.

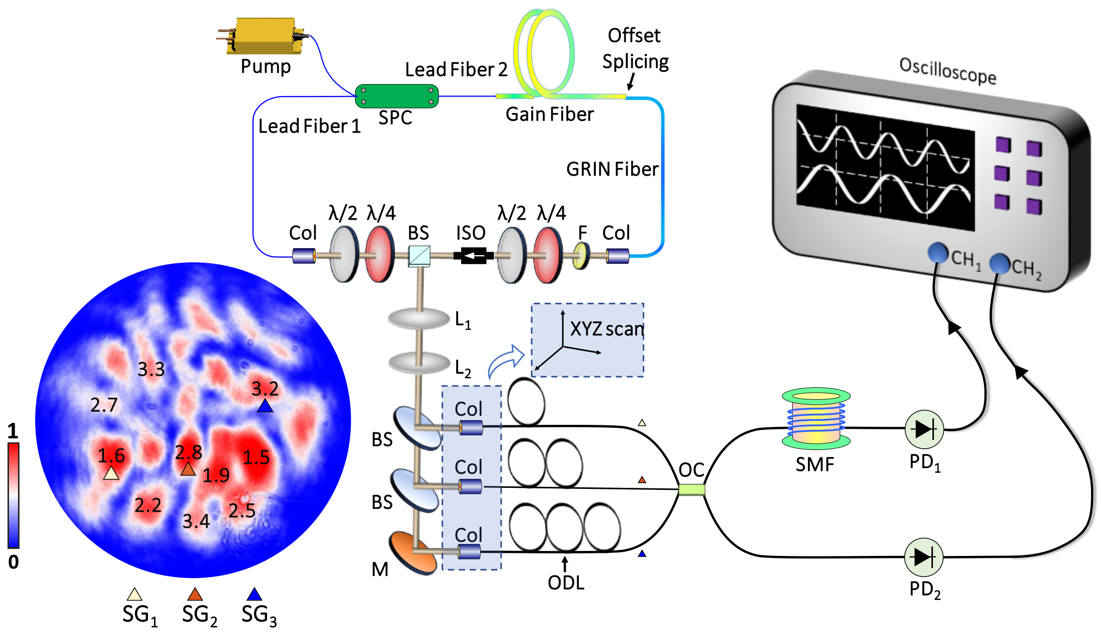

Supplementary Figure 1 | Full configuration of the experimental system. BS: beam splitter. Col: collimator. F: filter. ISO: isolator. L: lens. M: mirror. OC: optical coupler. ODL: optical delay line. PD: photodiode. SG: speckle grain. SMF: single-mode fiber. SPC: signal-pump combiner. λ/2: half-wave plate. λ/4: quarter-wave plate.

Supplementary Table 1: Estimated numbers of modes of the fibers used in the multimode fiber laser

**Fiber**	**Model number**	**Core NA**	**Core radius/μm**	**V number**	**Mode no.***
Lead fiber 1	Nufern LMA-GDF-15/130 0.08/0.46NA	0.08	7.5	3.5	6†
Lead fiber 2	Nufern LMA-GDF-15/130 0.08/0.46NA	0.08	7.5	3.5	6†
Gain fiber	Nufern LMA-YDF-15/130-VIII	0.08	7.5	3.5	6†
GRIN fiber	Thorlabs GIF625	0.275	31.25	51.0	650‡

*Counting the polarization; †Mode no. ≈ $V^2/2$; ‡Mode no. ≈ $V^2/4$

The beam size of the extracted laser is enlarged by a 5× magnification telescope composed of lenses L_1 and L_2 (30 mm and 150 mm focal lengths, respectively). The magnified laser beam is then launched to the MUST measurement system. In the MUST system, the laser signal is split by two BSs with ratios of 30:70 and 50:50, respectively. The signals of three speckle grains (SGs) in the multimode laser beam, as indicated in the left inset of **Supplementary Figure 1**, are individually received by three fiber collimators. The collected signals propagate through different optical delay lines (ODLs), which is subsequently combined by a 3×2 optical coupler (OC) for optical time division multiplexing (OTDM). The OTDM signal is split into two branches, one of which is directly detected by a high-speed photodiode (PD_2, 12 GHz). The other branch is launched to a long single-mode fiber (SMF, Nufern 1060-XP, 8 km length), which provides a large group velocity dispersion (GVD, about -0.3 ns/nm) for real-time spectroscopy through photonic time stretch. The time-stretched signal is detected by another high-speed PD (PD_1). The outputs of the PDs are finally recorded by a multi-channel real-time oscilloscope at a sampling rate of 80 GS/s.

Supplementary Note 2: Spatial mode profiles of the multimode fiber laser in different regimes.

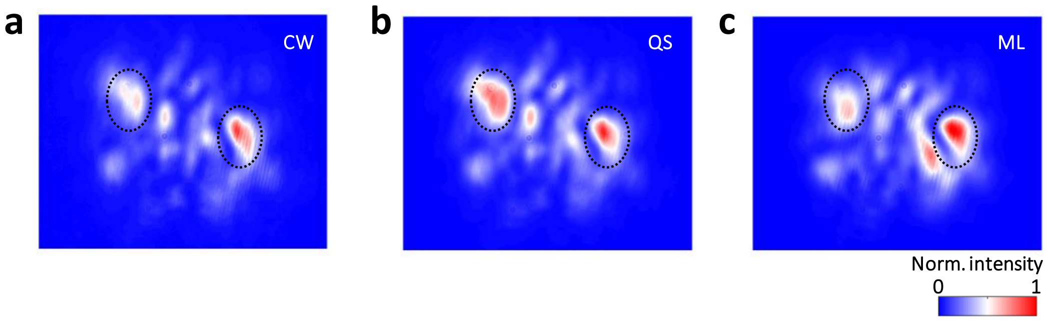

Supplementary Figure 2 | Spatial mode profiles of the multimode fiber laser. **a**. Continuous-wave (CW) regime. **b**. Q-switched (QS) regime. **c**. Mode-locking (ML) regime. The operating regime of the multimode fiber laser was changed by increasing the pump power, from 5 to 7 W in this case. The dotted circles indicate the intensity variation of the bright spots. The video record of the mode profile when increasing pump power is also provided in the **Supplementary Video 1**.

Please note that, the setting of the laser cavity in this measurement, mainly the state of polarization, is different from that of the inset of **Supplementary Figure 1**.

Supplementary Note 3: More details about the birth of the multipulse STML started from successive shockwaves.

Relaxation oscillation (RO): The RO is usually studied using a physical model that incorporates the time-dependent rate equations[1]. It was reported that the damping RO could turn into a sequence of raised RO spikes after introducing a saturable absorber (SA) effect in the 1D mode-locking (ML)[2]. In the 3D STML laser, in contrast, the MUST measurements show that the RO leads to two high-intensity shockwaves, as shown in the projections on the roundtrip-intensity plane of **Supplementary Figure 3a**. Please note that, there barely exist solitary pulses between the RO shockwaves, which can be considered as a signature of the RO that distinguishes itself from other states.

Q-switched mode-locking (QSML): The QSML involves both Q-switched modulation and mode-locked solitons. **Supplementary Figure 3a** highlights a QSML evolution that transits from the birth of multiple solitons to their annihilation, while **Supplementary Figure 3b,c** show the close-ups of the spectral and temporal evolutions in SG_1 (from RT 1700 to 2180). In the very beginning of the QSML state, multiple pulses appear on a broad background, which is a Q-switched pulse (QSP) in this case (**Supplementary Figure 3d**). These pulses interact with each other as they circulate in the cavity, and finally, the optimal pulse (denoted as "strong pulse" in **Supplementary Figure 3d**) survives while other subordinate ones are sacrificed due to the multi-dimensional gain competition and SA effect (**Supplementary Figure 3c**). This partially manifests the energy competition between the multiple solitons and their background (i.e., the QSP). In contrary to the RO state, the QSML results in regularly damping intensity spikes, as shown in the roundtrip-intensity projection of **Supplementary Figure 3a**.

Multipulse mode-locking (MPML): **Supplementary Figure 3e** highlights the transition from QSML to MPML, and **Supplementary Figure 4** shows the

corresponding temporal and spectral evolutions in a longer period. Complementary with **Fig. 2**, **Supplementary Figure 3f** shows the evolution of the field autocorrelation (FAC) trace calculated from the time-stretched waveforms in a truncated temporal span (i.e., the shaded area indicated in **Supplementary Figure 4b**), based on the Fourier transform method[3]. Here, the transition from a vibrating to "stable" multipulse regime is clearly illustrated (**Supplementary Figure 3f**). In the vibrating multipulse regime, there exist two sub-regimes: (***i***) and (***ii***). In (***i***), the intensity and time delay of the FAC sidelobes are both oscillating. Such an oscillating landscape implies the possibility of periodic energy exchanges between the solitons through transverse-longitudinal mode coupling. In (***ii***), these oscillation features are disappeared. Remarkably, it transits back to (***i***) again before directing to the "stable" multipulse regime, in which the FAC trace shows closer sidelobes (**Supplementary Figure 3g**).

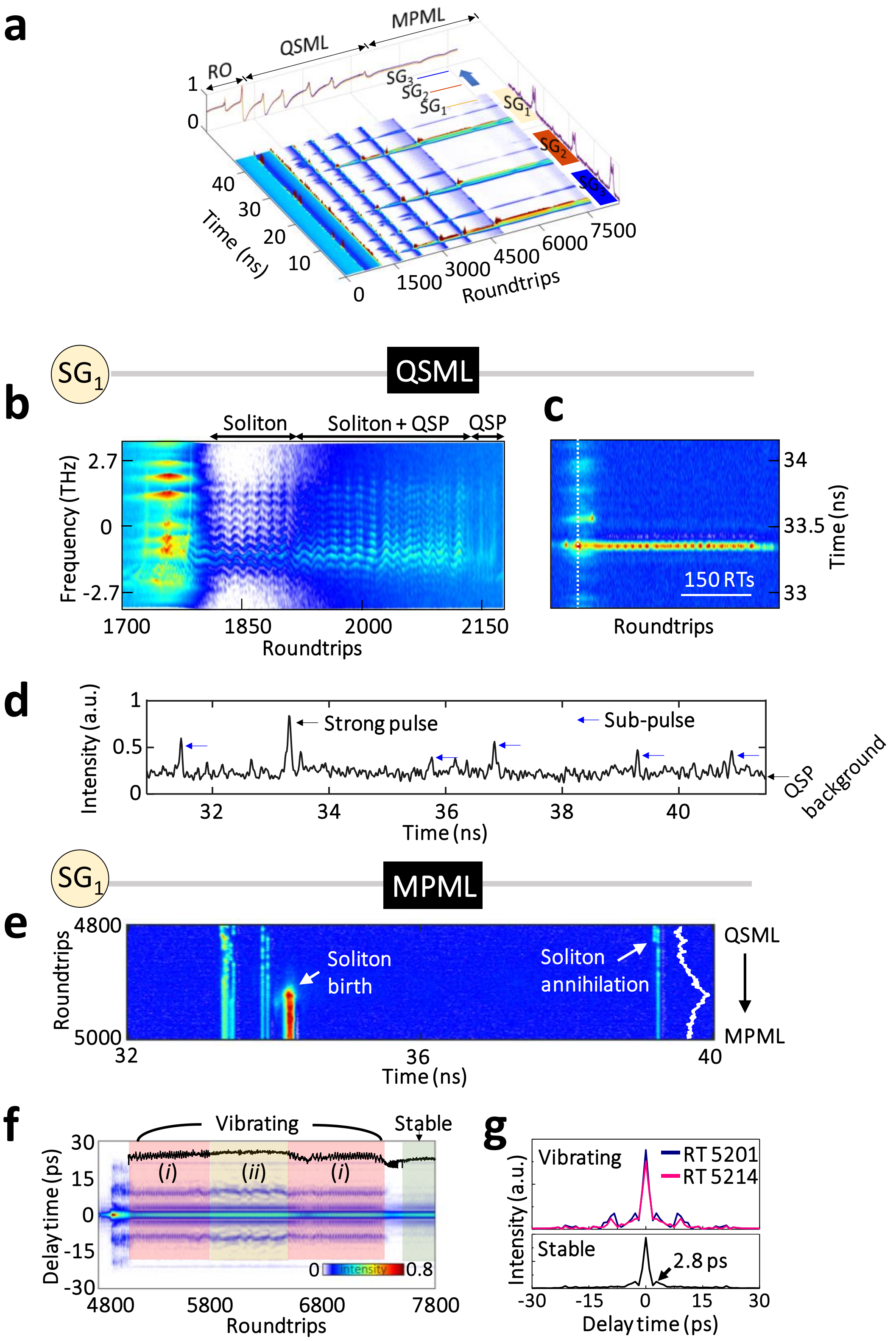

Supplementary Figure 3 | **Complementary information of the RO, QSML and MPML states**. **a**. OTDM spectral evolution of the multipulse STML birth. The projections on the roundtrip-intensity plane depict the total energy variations in different speckle grains ($SG_{1\text{-}3}$), while the curve on the time-intensity plane is the waveform at RT 7800. **b**–**d**. Details of the QSML in SG_1. **b** and **c** show the close-ups of

spectral and temporal evolutions, respectively. **d** illustrates a representative temporal waveform at RT 1760 (indicated by the dashed line in **c**), wherein the strong (main) pulse, subordinate pulses, and QSP background are indicated. **e**–**g**. Details of the MPML in SG_1. **e** illustrates the temporal transition from QSML to MPML, where the white curve on the right side shows the evolution of total energy. The evolution of the field autocorrelation (FAC) trace is shown in **f**, where the black curve on top presents the intensity evolution of the FAC sidelobes. **g** shows the typical FAC traces in the vibrating regime (top panel) and the stable regime (bottom panel).

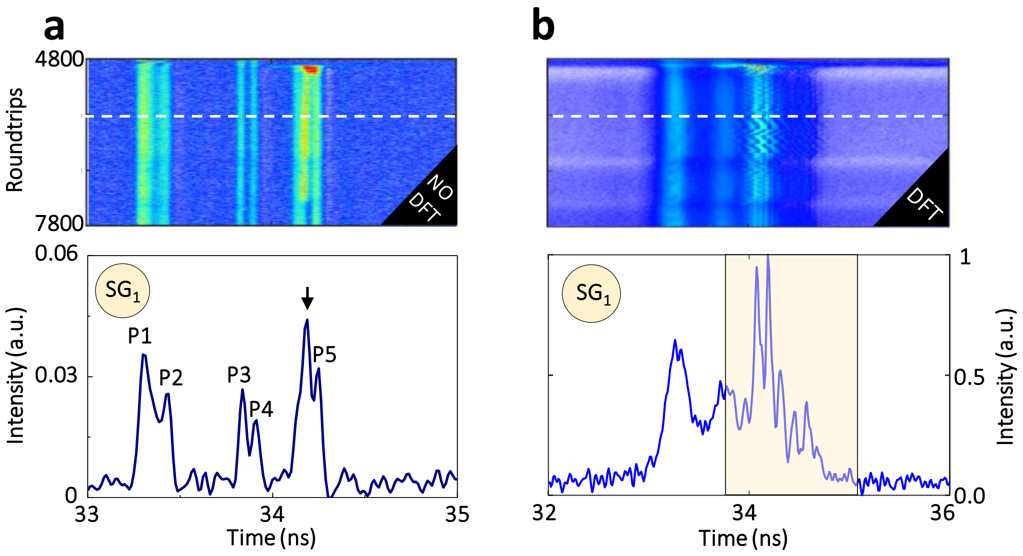

Supplementary Figure 4 | Spectral-temporal evolutions from QSML to MPML state in SG_1. a. Temporal evolution. **b**. Spectral evolution. Top panels: Real-time measurements of the evolutions. Bottom panels: Representative waveforms at RT 5800 (marked by the dashed lines). The pulse used for the FAC analysis is marked by the black arrow in **a**, and its temporal separation with respect to the others (namely, P1 to P5) ranges from 110 ps (to P5) to 880 ps (to P1). The truncated temporal span of 33.7 – 35.0 ns is extracted for the FAC calculation, as indicated by the shaded rectangle in **b**.

Supplementary Note 4: Photonic time stretch

4.1. Multipulse time stretch

The photonic time stretch technology, also known as dispersive Fourier transform (DFT), is a powerful tool for real-time spectroscopy of soliton dynamics. So far, extensive efforts have been devoted to studying the spectral dynamics of bound solitons using the photonic time stretch technology. Here, we consider a general situation with two pulses,

$$U_0(t) = u_1(t) + u_2(t-\tau)e^{-i\Delta\omega t}, \qquad (S1)$$

where, τ and $\Delta\omega$ are the temporal separation and central frequency difference, respectively. After performing photonic time stretch, the field envelope becomes

$$U(t) = \mathfrak{I}^{-1}\{\mathfrak{I}[U_0(t)]exp(iD_2\omega^2/2)\}, \qquad (S2)$$

where, D_2 is the chromatic dispersion used in the photonic time stretch. By applying Fourier transform to $U_0(t)$, Eq. $(S2)$ can be rewritten as

$$U(t) = \mathfrak{I}^{-1}\{[\tilde{u}_1(\omega) + \tilde{u}_2(\omega-\Delta\omega)e^{-i(\omega-\Delta\omega)\tau}]exp(iD_2\omega^2/2)\}. \qquad (S3)$$

By further exploiting the stationary-phase approximation, $U(t)$ becomes

$$U(t) = \sqrt{\frac{-2\pi}{-iD_2}}e^{-i\frac{t^2}{D_2}}\left[\tilde{u}_1\left(\frac{t}{-D_2}\right) + \tilde{u}_2\left(\frac{t-\tau}{-D_2} - \Delta\omega\right)e^{i\Delta\omega\tau - i\frac{\tau^2}{2D_2}} \cdot e^{i\frac{\tau}{D_2}t}\right]. \qquad (S4)$$

Eq. $(S4)$ shows that, the time-stretched waveform exhibits additional features that oscillate at a frequency of D_2/τ. It is also worth noting that the presence of $\Delta\omega$ results in a relative phase shift of the oscillation.

4.2. Spectral overlapping of multipulse time stretch

In the multipulse scenario, spectral overlapping can occur after the photonic time stretch, particularly when the pulses are closely located. Regarding this potential issue, we here investigate a pulse pair with varying temporal separation. As shown in **Supplementary Figure 5a**, different circumstances are considered: ***i***) a pulse pair with small temporal separation (similar to the bound solitons or soliton-molecule) that produces resolvable spectral interference fringes, i.e., oscillating in a long period (**Supplementary Figure 5b**). In this case, the time-stretched waveforms highly overlap and interfere with each

other at a low fringe frequency, which is inversely proportional to the temporal separation; ***ii***) a pulse pair with large temporal separation that results in invisible interference fringes under time-stretched spectroscopy, i.e., beyond the spectral resolution of the time-stretched spectroscopy. In this situation, the pulses are partially overlapped, and the interference process evolves due to the imparted frequency chirp (**Supplementary Figure 5c**); ***iii***) a pulse pair is widely separated, such that there is no overlapping between the time-stretched waveforms.

Specifically, the case of ***ii***) can be written as

$$\left|\frac{\lambda^2 D_2}{c\Delta t}\right| < T_{sep} < \left|D_2\Omega_p\right|, \qquad (S5)$$

where, Δt is the temporal resolution of the detection system, λ is the central wavelength, c is the speed of light, and Ω_p is the spectral width of the optical pulses. The key parameters in consistence with the experiments are $\Delta t = 20\, ps$, $\Omega_p = 5\, nm$ and $D_2 = -300\, ps/nm$, and we have

$$57\, ps < T_{sep} < 1520\, ps. \qquad (S6)$$

We illustrate the temporal waveforms for cases ***i***) (**Supplementary Figure 5b**) and ***ii)*** (**Supplementary Figure 5c**). In ***i***), as expected, the spectral interference fringes are visible at a low temporal resolution (**Supplementary Figure 5b**), mimicking a practical resolution in the experiments. The sidelobes of its Fourier transform (FT) manifest as a bell-shaped envelope without any fine structures (**Supplementary Figure 5d**). In ***ii***), in contrast, it is unable to resolve the interference fringes at a low temporal resolution (**Supplementary Figure 5c**), while the corresponding FT sidelobes exhibit satellite peaks (**Supplementary Figure 5e**). As a result, the coexistence of multiple pulses with moderate temporal separations, i.e., Eq. $(S6)$, can be partially investigated using photonic time stretch and certain important information can still be extracted, as showcased in **Supplementary Figure 5f**.

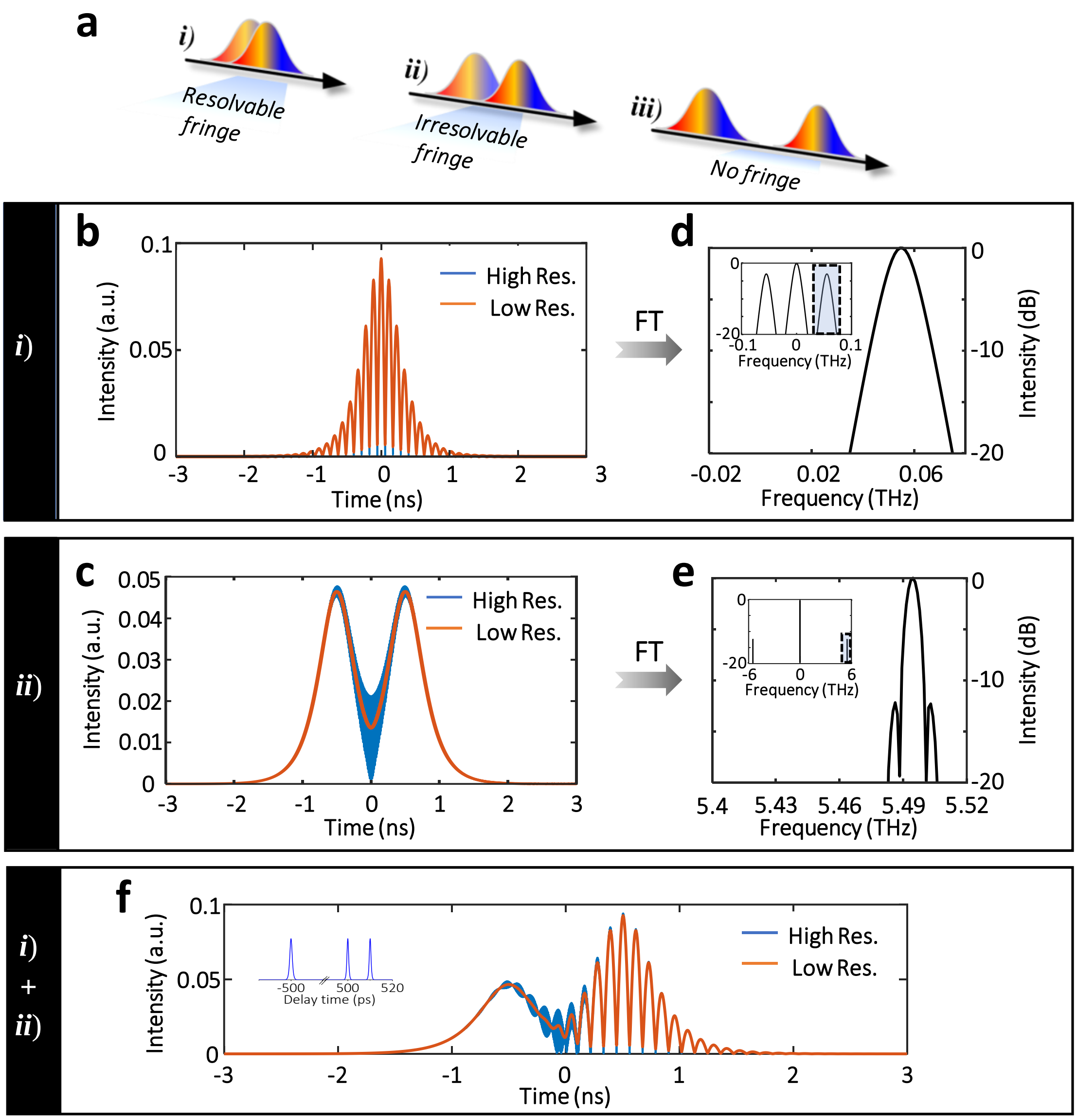

Supplementary Figure 5 | Time-stretched characteristics of pulse pairs with different temporal separations. **a**. Representative circumstances of the time-stretched process with different temporal separations. **b**,**c**. Pulse pairs with temporal separation of 10 ps and 1 ns, i.e., corresponding to cases ***i***) and ***ii***), respectively. Each waveform is sampled at both high (blue) and low (orange) temporal resolutions, i.e., 90 fs and 9 ps, respectively, and the latter one is similar to the sampling period of 12.5 ps in the experiments (i.e., a sampling rate of 80 GS/s). **d**,**e**. Fourier transforms (FT) of the high-resolution waveforms (blue) in **b**,**c**. Here, the FT sidelobes are magnified to identify the difference, while the full FT waveforms are shown as insets. **f**. Time-stretched waveforms of a three-pulse cluster. The separations between the adjacent pulses are 10 ps and 1 ns, respectively, as shown in the inset.

Supplementary Note 5: More details about the birth of the multipulse STML started from a single shockwave.

Supplementary Figure 6a illustrates the full dataset of the MUST measurement of the second shot of multipulse STML birth started from a single shockwave. In contrast to the first shot, here the generated multipulse pattern exhibits irregular arrangement, and the maximum separation between the pulses is larger than the time delays set for different speckle grains in the OTDM. Thus, the crosstalk among the speckle grains occurs in this case. However, the key information can be largely recovered using the preset OTDM time delays of different speckle grains, i.e., 15.06 ns and 30.75 ns for SG_1–SG_2 and SG_1–SG_3, respectively. The visible pulse separations of the pulse pairs P_1 and P_2 are 550 and 240 ps, respectively. According to **Supplementary Note 4**, no measurable interference fringe corresponding to these two temporal separations is expected in the time-stretched measurement. As a result, the measured interference fringes of P_1 (**Supplementary Figure 6d**, also see **Fig. 3c**) should have been generated by bound pulses with much closer separations, which is unresolvable in the temporal domain (**Fig. 3a**).

The FAC technology is applied to explore more details about the interference fringe pattern of P_1 (**Supplementary Figure 6d**). **Supplementary Figure 7a** is the evolution of the FAC trace, wherein the behavior of the symmetric sidelobes manifests the dynamics of the involved bound solitons. The variations of the relative peak intensity and time delay of the FAC sidelobe, marked by the black arrow in **Supplementary Figure 7a**, are analyzed as **Supplementary Figure 7b**. As can be observed, the stable FAC sidelobe is established after about 620 RTs, as indicated by the red arrow in **Supplementary Figure 7b**. Based on the FAC analysis, we can imagine that the pulse cluster P_1 with compound structures may be composed of soliton-molecules and other well-separated pulses, as showcased in **Supplementary Figure 8**.

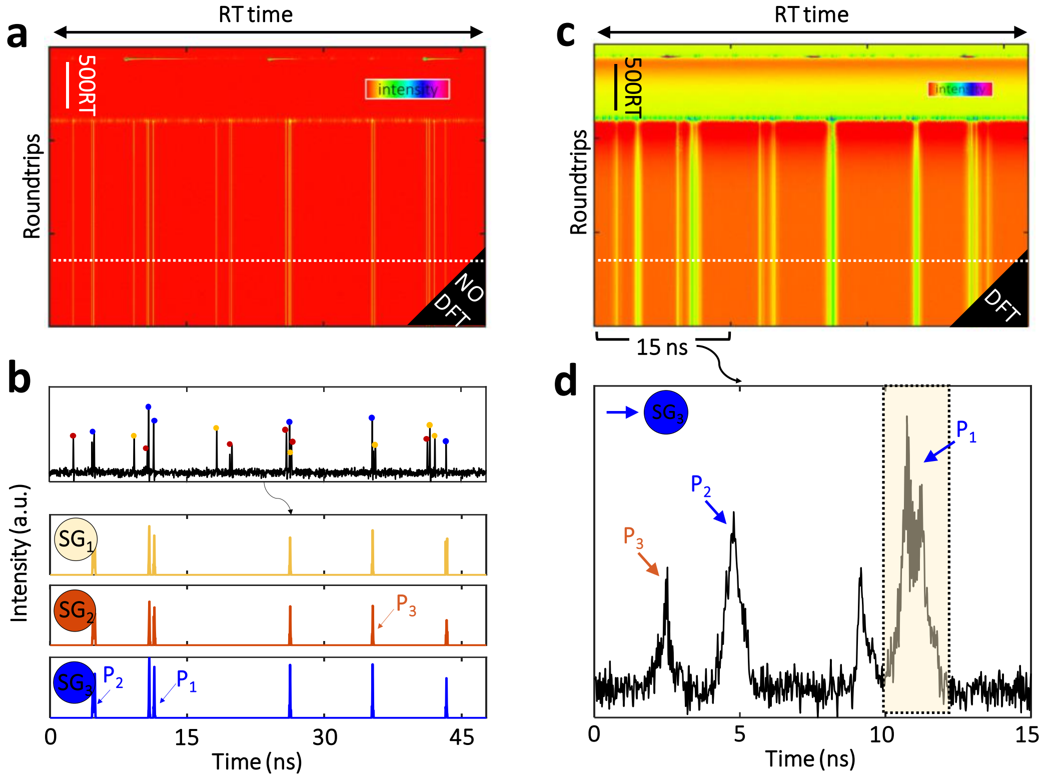

Supplementary Figure 6 | Temporal and spectral evolutions of the multipulse STML birth started from a single shockwave. **a**. Temporal evolution. **b**. Snapshot of the temporal evolution, as indicated by the dashed line in **a**. Top panel is the OTDM signal of speckle grains $SG_{1\text{–}3}$, and the bottom ones are the demultiplexed temporal pulse patterns for different speckle grains. **c**. Spectral evolution. **d**. Snapshot of the spectral evolution, as indicated by the dashed line in **c**. Pulse pairs labeled by P_1, P_2 and P_3 in the main context are also denoted in **b** and **d**. The FAC calculation is performed for the area indicated by the shaded area in **d**, see **Supplementary Figure 7**.

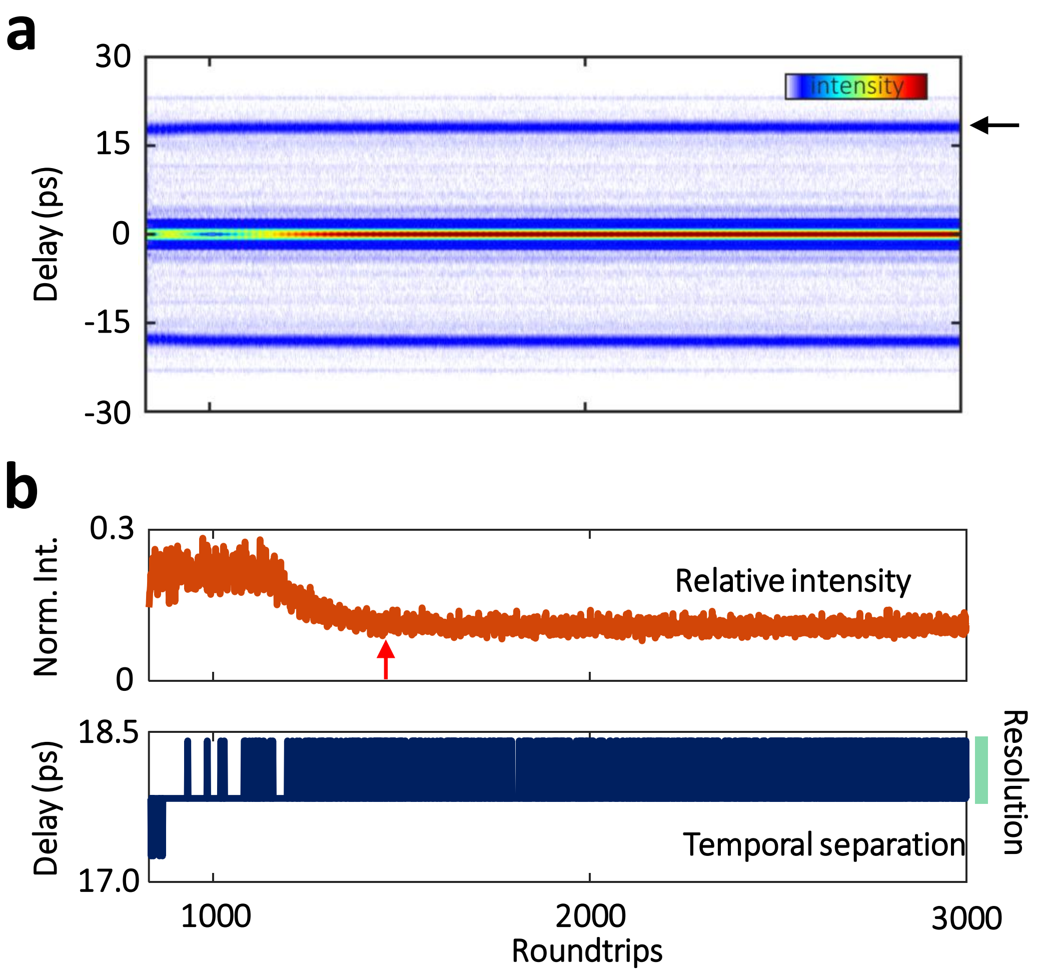

Supplementary Figure 7 | FAC analysis of the spectral interferogram of P_1. a. FAC evolution. The arrow indicates the secondary peak that reveals the features of the temporally unresolved pulses. **b**. Relative peak intensity (top) and time delay (bottom) evolutions of the secondary peak indicated in **a**.

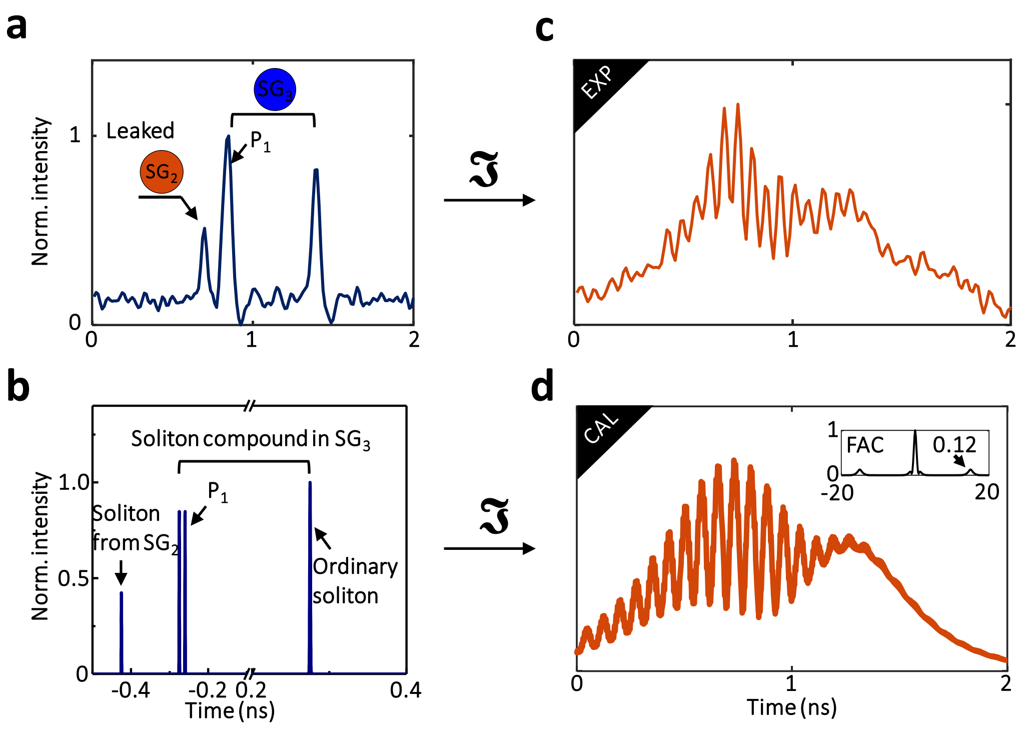

Supplementary Figure 8 | Pulse cluster P1. a,b. Temporal waveforms of the measurement and calculation respectively. **c,d**. Spectral waveforms of the measurement and simulation, respectively.

Supplementary Note 6: Vibrating characteristics of 3D soliton-molecules

The relative phase and temporal separation are two important parameters for characterizing the coherent pulse pair (i.e., the soliton-molecule in this case) that affect the generated interference fringe pattern. The change of either one results in the variation of the spectral interferogram. As shown in **Supplementary Figure 9a**, the center wavelength of the modulated spectrum in SG_1 maintains consistent over roundtrips, while it experiences obvious change in SG_2 (**Supplementary Figure 9d**). The relative phase evolution of the soliton-molecule can be retrieved from the spectral interferograms using the method described in **Section 7**, and the results are shown in **Supplementary Figures 9b,e**. A phenomenological function[4] can be applied to fit the results,

$$\Delta\varphi(R) = aRT + A\sin\left[\frac{2\pi(RT - RT_0)}{T}\right] + \varphi_0, \qquad (S7)$$

where, RT is the roundtrip number, a is the slope. A, RT_0 and T are the amplitude, offset and period of the sinusoidal part, respectively. φ_0 is the bias. The key parameters used to fit the relative phase evolution are provided in **Supplementary Table 2**. The temporal separation of the soliton-molecule, on the other hand, manifests as the time delay of the FAC sidelobes, as shown in **Supplementary Figures 9c,f**.

Supplementary Table 2: Parameters used to fit the relative phase evolutions of the soliton-molecules

Speckle grain	a	A	RT_0	T	φ_0
SG_1	0	0.37	960	2018.8	-1.72
SG_2	-0.0054	0.225	301.8	1095.4	-0.6

The asymmetry of the soliton-molecule, mainly the relative intensity and temporal separation, can largely influence the visibility of the interference fringe (**Supplementary Figure 10**). Thus, the shallow intensity modulation of the interference fringe pattern of **Fig. 4** and **Supplementary Figure 9** can be mainly attributed to the asymmetric intensities of the soliton pair.

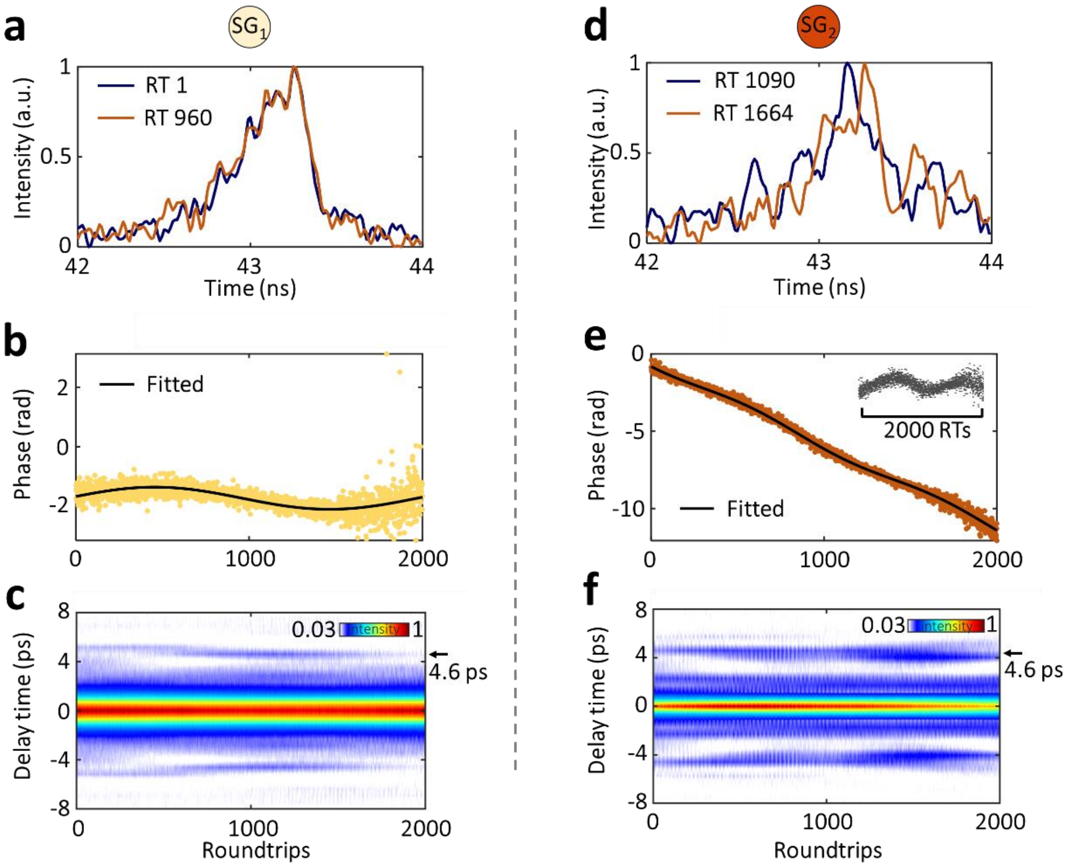

Supplementary Figure 9 | Features of the soliton-molecules in SG_1 and SG_2. a. Snapshots of time-stretched waveforms in SG_1. **b**. Relative phase evolution of the soliton-molecule in SG_1. **c**. Evolution of the FAC trace in SG_1. **d**–**f**. Corresponding measurements of SG_2. The fitted curves in **b** and **e** are calculated using the coefficients provided in **Supplementary Table 2**. The inset of **e** is the oscillation component.

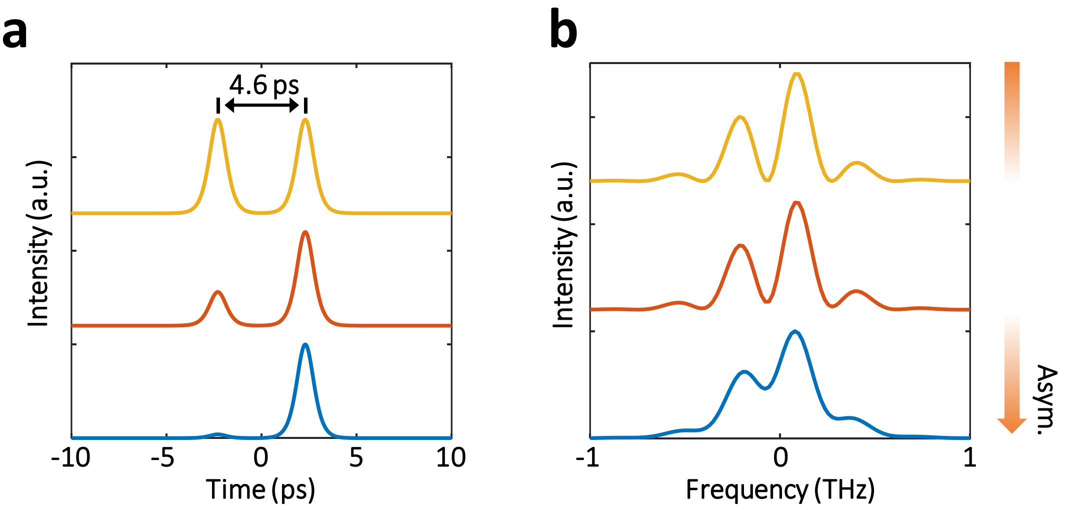

Supplementary Figure 10 | Spectral-temporal features of asymmetric soliton-molecules. Temporal (**a**) and spectral (**b**) profiles of soliton-molecules with varying relative intensity.

Supplementary Note 7: Calculation of relative phase between solitons in soliton-molecules

In addition to the temporal separation, the relative phase is another key characteristic of the soliton-molecule, which can be calculated from two maximum peaks of the spectral interferogram and the carrier frequency. However, it is challenging to precisely obtain the carrier frequency when many solitons are involved. Here, we explore a universal method[5] to retrieve the relative phase from the spectral interferogram of soliton-molecules.

First, the spectral intensity of a soliton-molecule can be expressed as

$$\begin{aligned}\tilde{I}(\omega) &= |\Im[E_1(t)+E_2(t-\tau)]|^2 \\ &= \left|\tilde{E}_1(\omega)\right|^2 + \left|\tilde{E}_2(\omega)\right|^2 + \tilde{E}_1(\omega)\tilde{E}_2^*(\omega)e^{i\omega\tau} + \tilde{E}_1^*(\omega)\tilde{E}_2(\omega)e^{-i\omega\tau}, \qquad (S8)\end{aligned}$$

where, $\Im$ denotes the FT operation, and ~ represents the result of the FT operation. For convenience, we rewrite Eq. ($S8$) as

$$\tilde{I}(\omega) = \tilde{e}_0(\omega) + \tilde{e}_1(\omega)e^{i\omega\tau} + \tilde{e}_{-1}(\omega)e^{-i\omega\tau}, \qquad (S9)$$

where

$$\tilde{e}_0(\omega) = \left|\tilde{E}_1(\omega)\right|^2 + \left|\tilde{E}_2(\omega)\right|^2,$$

and $\tilde{e}_1(\omega)$, i.e., the complex conjugate of $\tilde{e}_{-1}(\omega)$, is given by

$$\tilde{e}_1(\omega) = \tilde{E}_1(\omega)\tilde{E}_2^*(\omega).$$

Without loss of generality, the relative phase $\Delta\varphi$ in the frequency domain can be defined as

$$\Delta\varphi = \sphericalangle[\tilde{E}_1(0)\tilde{E}_2^*(0)]. \qquad (S10)$$

Then, the intensity function $\tilde{I}(\omega)$ is inversely Fourier transformed, and becomes

$$I(t) = e_0(t) + e_1(t+\tau) + e_{-1}(t-\tau). \qquad (S11)$$

The function $e_1(t+\tau)$ is readily obtained by isolating the sidelobes of $I(t)$ located at $t = -\tau$, and it is then Fourier transformed to extract the value of $\Delta\varphi$,

$$\Delta\varphi = \sphericalangle\Im[I(t)H(t+\tau)]|_{\omega=0} = \sphericalangle\Im[e_1(t+\tau)]|_{\omega=0}, \qquad (S12)$$

where, $H(t+\tau)$ is the time gating function at $t = -\tau$. For the prerequisite of $\omega = 0$, it is not necessary to temporally shift $e_1(t+\tau)$ by τ before performing FT. To validate the proposed method, numerical studies are performed and the results are shown in **Supplementary Figure 11**, which exhibits a good agreement with the preset. It is worth noting that this method is valid for any carrier frequencies — offering a general means to evaluate the relative phase of the soliton-molecule.

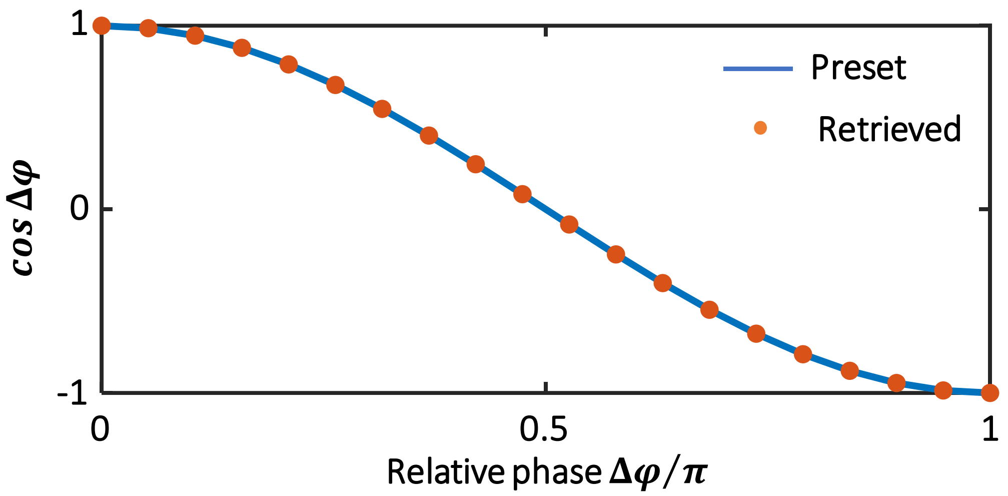

Supplementary Figure 11 | Retrieved relative phase using the proposed method (dotted line) and the preset value (solid line).

Supplementary Note 8: Additional data for the internal breathing dynamics

The intensities of the pulses in SG_1 oscillate at a high frequency, while a low frequency for SG_3, as shown in **Supplementary Figure 12**. The corresponding intensity integration evolutions in the temporal domain exhibit similar features as their intensities (**Supplementary Figure 13**). In comparison to the internal breathing phenomena

shown in **Fig. 5**, we found other internal breathing dynamics with dual-period pulsation, as showcased in **Supplementary Figure 14**.

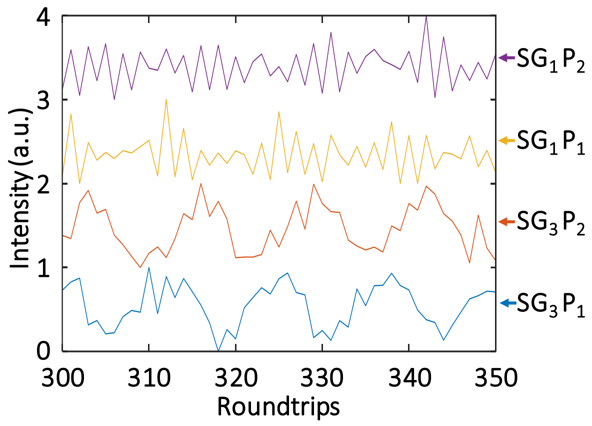

Supplementary Figure 12 | Intensity evolutions of the pulses in the temporal domain, i.e., left panels of Fig. 5e,f. Here, the curves have been vertically offset for better visualization.

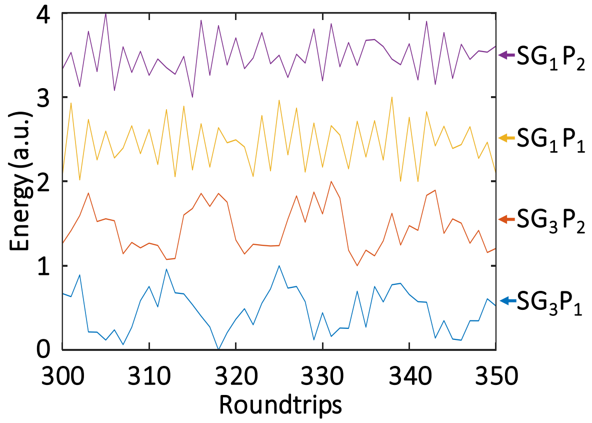

Supplementary Figure 13 | Intensity integration evolutions of the pulses in the temporal domain, i.e., left panels of Fig. 5e,f. Here, the curves have been vertically offset for better visualization.

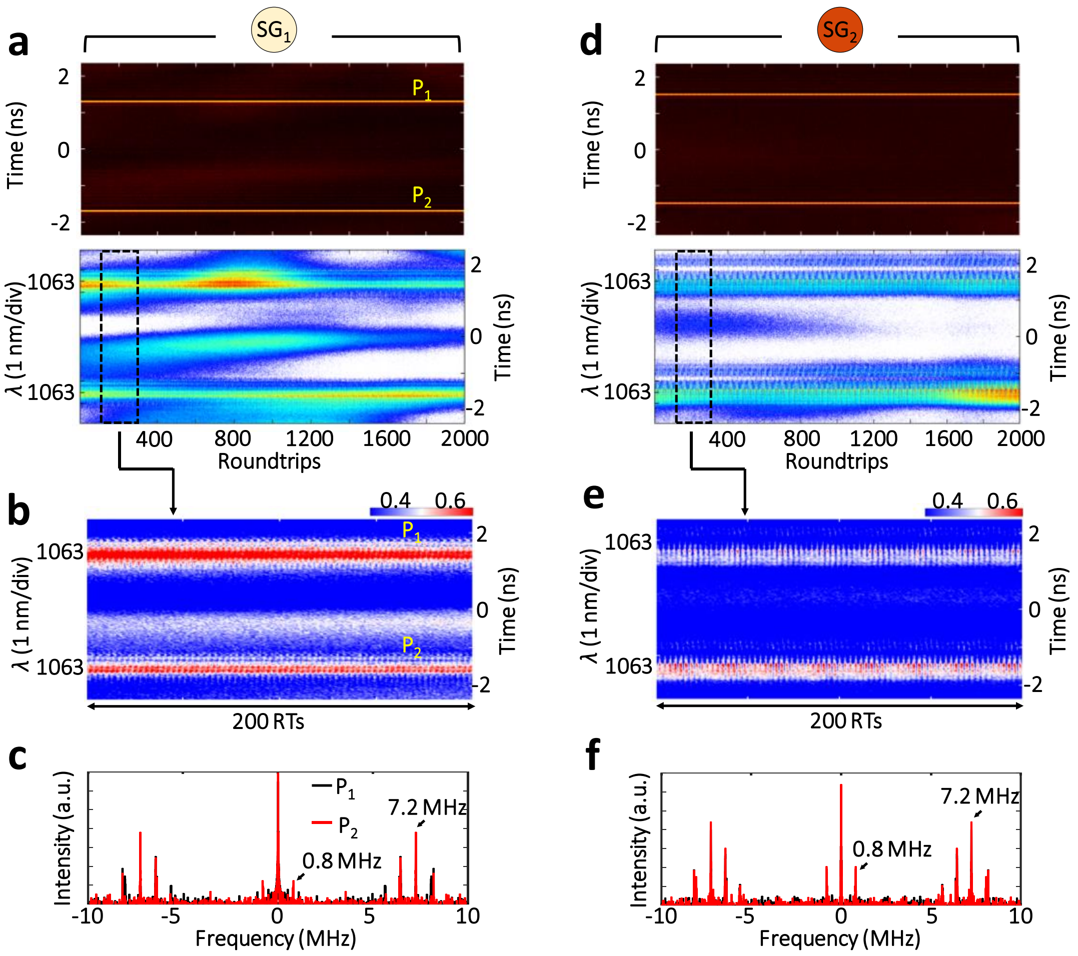

Supplementary Figure 14 | Internal breathing dynamics of 3D solitons with dual-period pulsation. **a–c**. Evolutionary behaviors in SG_1. **a**. Temporal (top) and spectral (bottom) evolutions. **b**. Close-up of spectral breathing over 200 RTs. **c**. Fourier transform of the peak intensity of **b**, where two oscillation frequencies of 7.25 and 0.81 MHz are clearly shown. **d–f**. Cases in SG_2. Note that the oscillation frequencies are consistent with that of SG_1.

Supplementary Note 9: 3D numerical studies

In our numerical studies, we consider a ring laser cavity that consists of a GRIN gain fiber (50 cm length, 62.5 μm core size), an artificial saturable absorber, a beam splitter, a bandpass filter, and a space filter. Note that, to reduce the calculation time, the simulation condition of the laser cavity has been simplified from the experimental setup, mainly combining all different fibers of the experimental laser cavity into a single GRIN gain fiber and using a saturable absorption function for STML.

A numerical model of the STML laser has been proposed in recent theoretical work[6]. We adopt this numerical model in our numerical investigation. In brief, the propagation of the multimode light field in the GRIN gain fiber is described by the generalized multimode nonlinear Schrödinger equations (GMMNLSEs)[7], i.e.,

$$\partial_z A_p(z,t) = i\delta\beta_0^{(p)} A_p - \delta\beta_1^{(p)} \partial_t A_p + \sum_{m=2}^{3} i^{m+1} \frac{\beta_m^{(p)}}{m!} \partial_t^m A_p + i\frac{n_2\omega_0}{c} \sum_{l,m,n}^{N} S_{plmn}^K A_l A_m A_n^* , \quad (S13)$$

where, $A_p(z,t)$ is the electric field of the spatial mode p, $\delta\beta_0^{(p)}$ and $\delta\beta_1^{(p)}$ are the propagation constant and group velocity of the spatial mode p, $\beta_m^{(p)}$ is the *m*-order dispersion coefficient. n_2, ω_0, and c are the nonlinear refractive index, center radial frequency and speed of light in vacuum, respectively. S_{plmn}^K is the nonlinear coupling coefficient of the Kerr effect.

Then, the 3D light field gain of the GRIN gain fiber, as described in ref. 6, can be rewritten as,

$$g(x,y,\omega) = \frac{g_0(\omega)}{1 + \int |A(x,y,t)|^2 dt / F_{sat}}, \quad (S14)$$

where, F_{sat} is the saturation fluence (0.3 nJ/μm^2 in this work), g_0 is the small signal gain coefficient, i.e., 35 m^{-1} here. The saturable absorption effect is established using an ideal transfer function after the gain fiber propagation, i.e.,

$$A(x,y,t) \to A(x,y,t)\sqrt{1 - \left(1 + |A(x,y,t)|^2 / I_{sat}\right)^{-1}}, \quad (S15)$$

where, I_{sat} is the saturation intensity, which is set to be 50 GW/cm^2.

The oscillation signal is extracted with a constant ratio, i.e.,

$$A(x,y,t,z) \to A(x,y,t,z)\sqrt{0.4}\,. \quad (S16)$$

In the simulation, for a moderate calculation time, only 10 transverse modes are considered without loss of generality. The second-order dispersions of these modes used in the numerical studies are shown in **Supplementary Figure 15**. After successfully mode-locking, a typical intensity distribution among these modes is shown in **Supplementary Figure 16**, while the evolutions of their intensity distributions at different wavelengths are provided in **Supplementary Figure 17**.

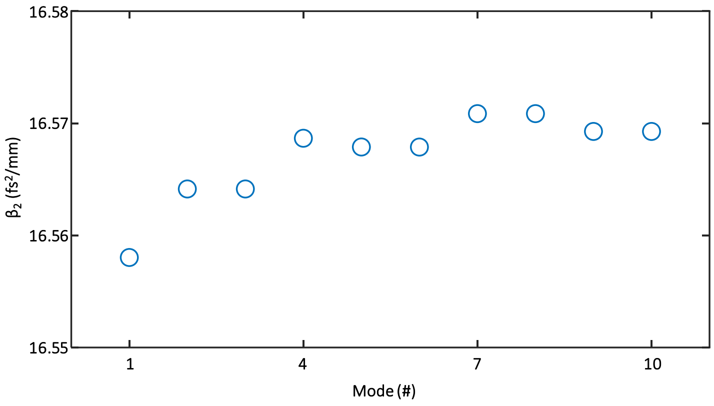

Supplementary Figure 15 | Second-order dispersion of the GRIN fiber. Here, the first 10 propagating modes are considered at 1063 nm.

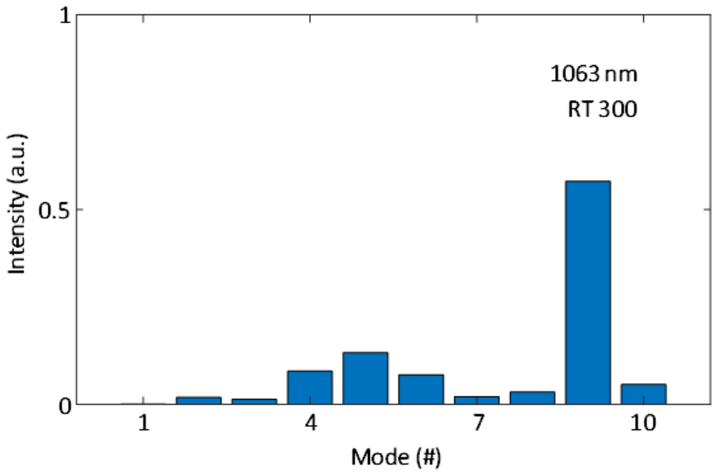

Supplementary Figure 16 | Intensity distribution among modes at RT 300. Here, the wavelength is 1063 nm.

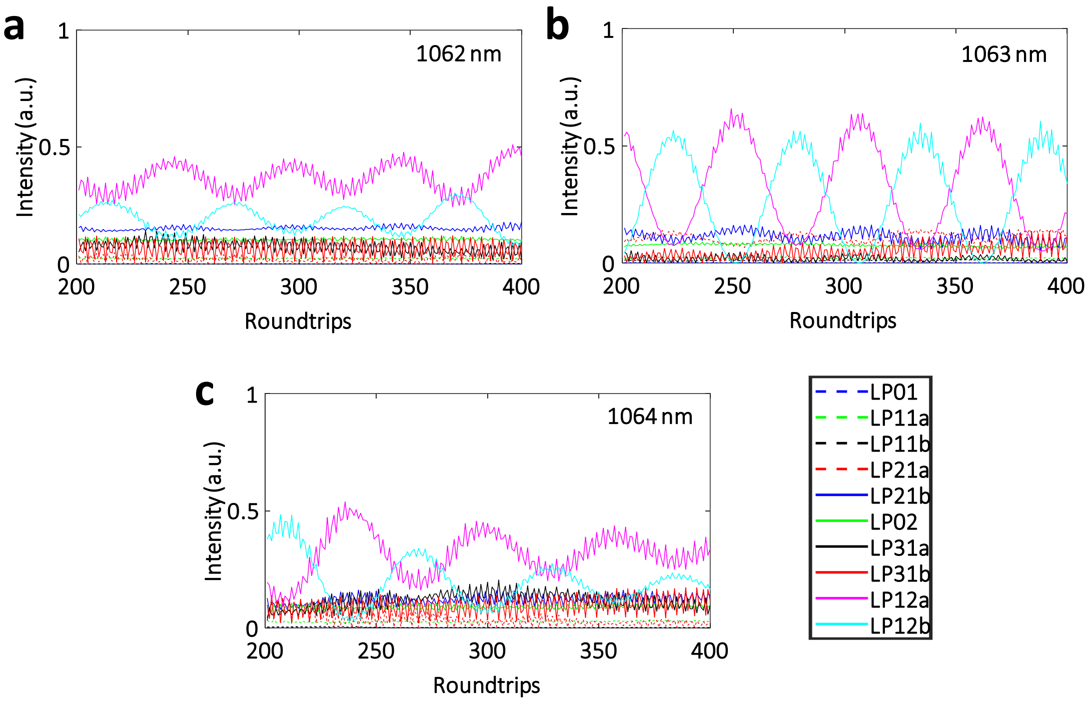

Supplementary Figure 17 | Evolutions of the intensity distributions. a. 1062 nm. **b**. 1063 nm. **c**. 1064 nm.

As shown in **Supplementary Figure 18**, two asynchronously pulsating pulses are generated, i.e., P_1 and P_2 (**Supplementary Figure 18b**). Please note that, to mimic the modal dispersion, a time delay is applied to the pulses of the degenerate transverse modes, i.e., the groups of {LP_{11a}, LP_{11b}} and {LP_{21a}, LP_{21b}}. The simulation results clearly show that the co-propagation of time-delayed pulses in intrinsic degenerate modes can generate the spectral-temporal dynamics analogous to the experimental observation (**Fig. 5**).

As illustrated in **Supplementary Figure 19**, internal spatiotemporal transient dynamics of the 3D solitons are recognized, where the transverse mode rotating and time delay between the degenerate modes play important roles. To understand the physical origin, we refer to the minimum loss principle (or the maximum gain extraction)[6], and a qualitative understanding can be intuitively obtained from the gain function Eq. ($S14$). The pulse energy tends to be equally distributed over the spatial modes, otherwise, a strong gain saturation can be expected. If there is no time delay between the degenerate

modes, higher peak power is generated, which in turn leads to the destructive wave collapse[8]. Therefore, to obtain the maximum gain extraction without wave destruction, a hybrid form of 3D solitons with both internal modal variation and time offset is favorable. In addition to the internal spatiotemporal dynamics within 3D solitons, pulse-to-pulse dynamics have also been observed when the multimode fiber laser operates in the partial-STML regime, as shown in **Supplementary Figures 20,21**. These numerical results suggest that single-shot characterization technologies are especially important for unveiling the dynamics of STML multimode fiber lasers and understanding their physical origins.

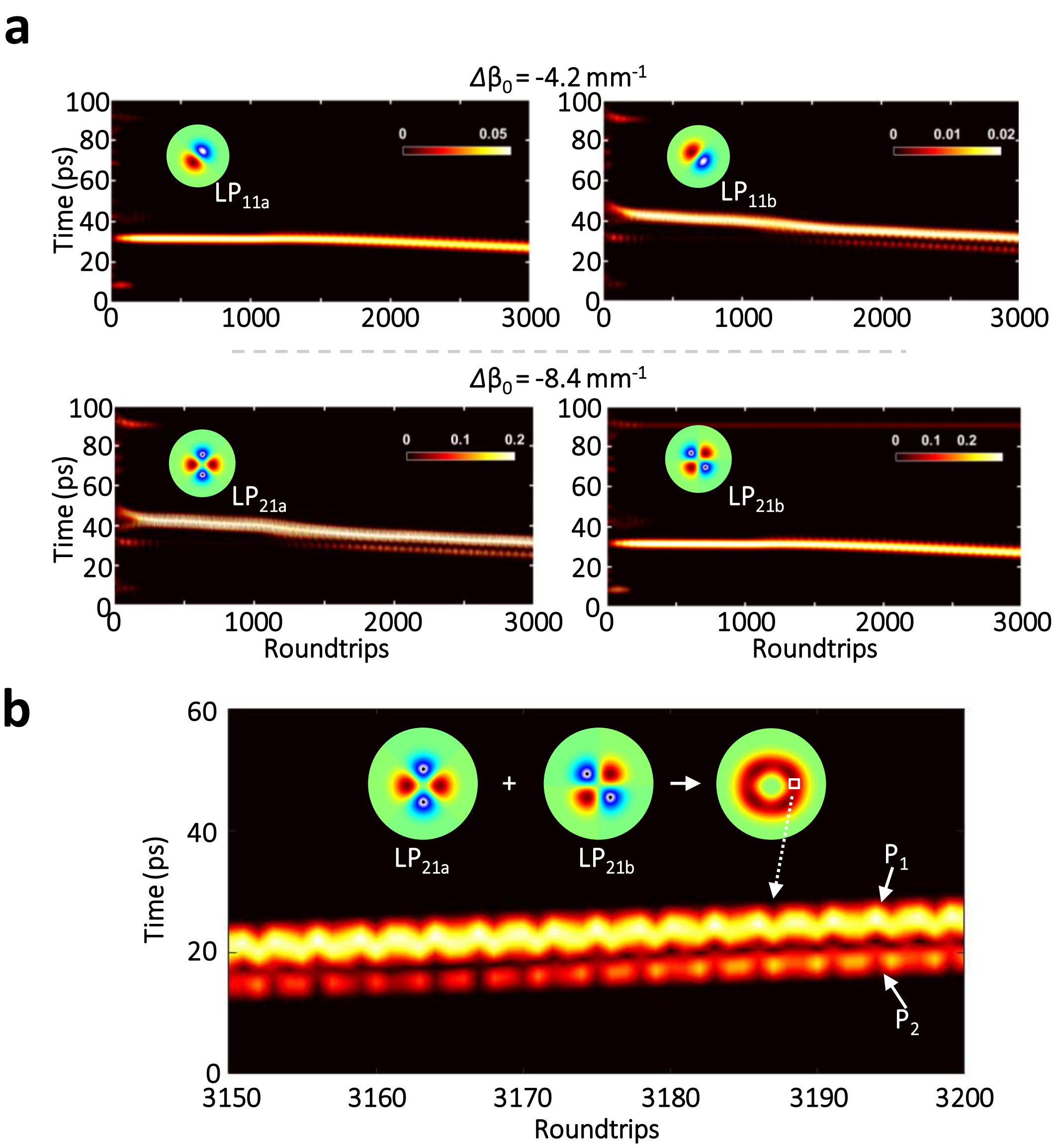

Supplementary Figure 18 | **Simulated temporal evolutions of different transverse modes**. **a**. Temporal evolutions of the LP_{11} and LP_{21} modes. **b**. Temporal evolution at the spatial location (white square) of the two-mode soliton (LP_{21a} and LP_{21b}). Here, two asynchronously pulsating pulses (P_1 and P_2) are generated.

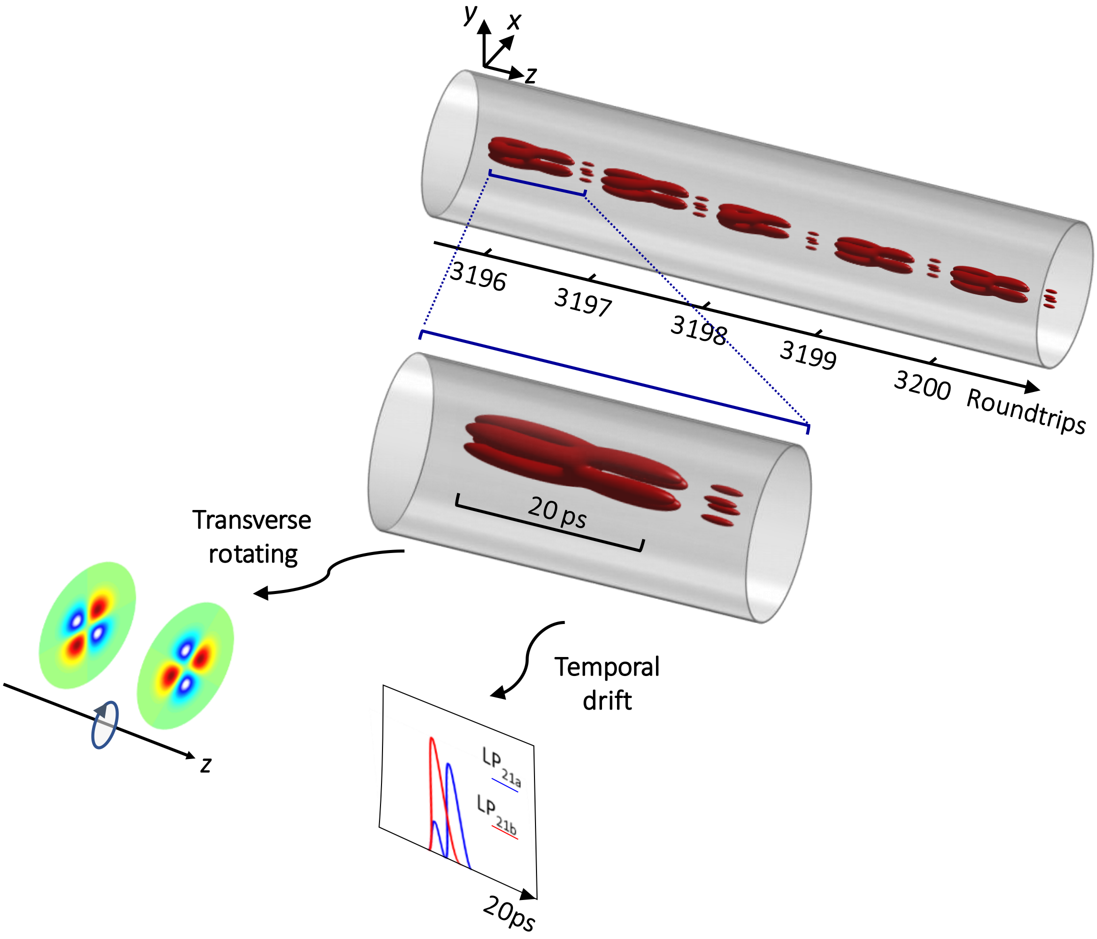

Supplementary Figure 19 | **3D pulse train composed of LP_{21a} and LP_{21b} modes**. Please note that, the time interval between pulses, i.e., the roundtrip time, has been deliberately shortened for better visualization. Insets show the transverse mode rotating and temporal drift.

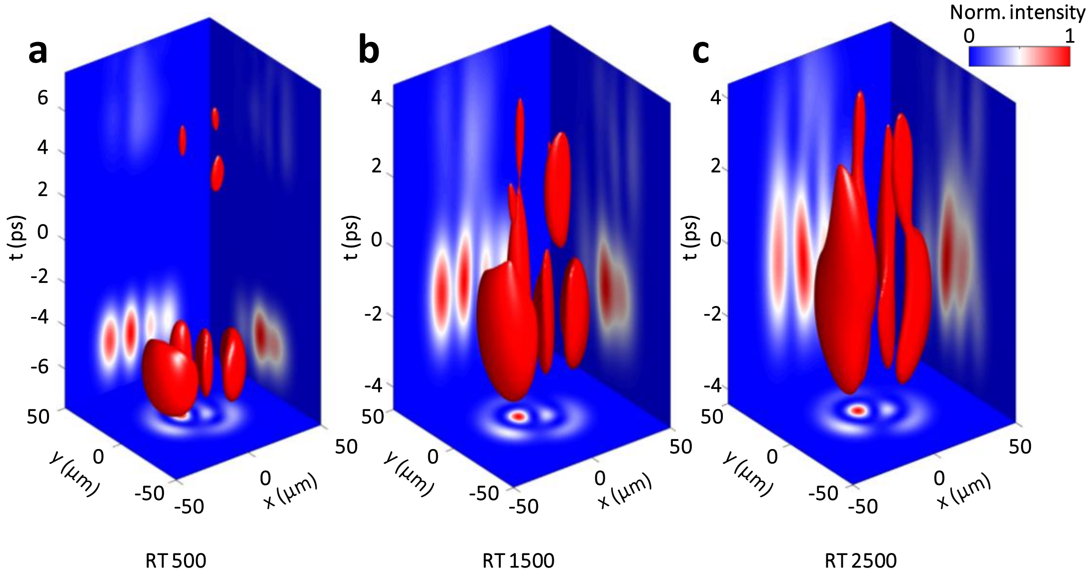

Supplementary Figure 20 | **Spatiotemporal dynamics in the numerical simulation**. **a**. RT 500. **b**. RT 1500. **c**. RT 2500. Here, the 3D isosurface plots are set to 10% of the peak field intensity.

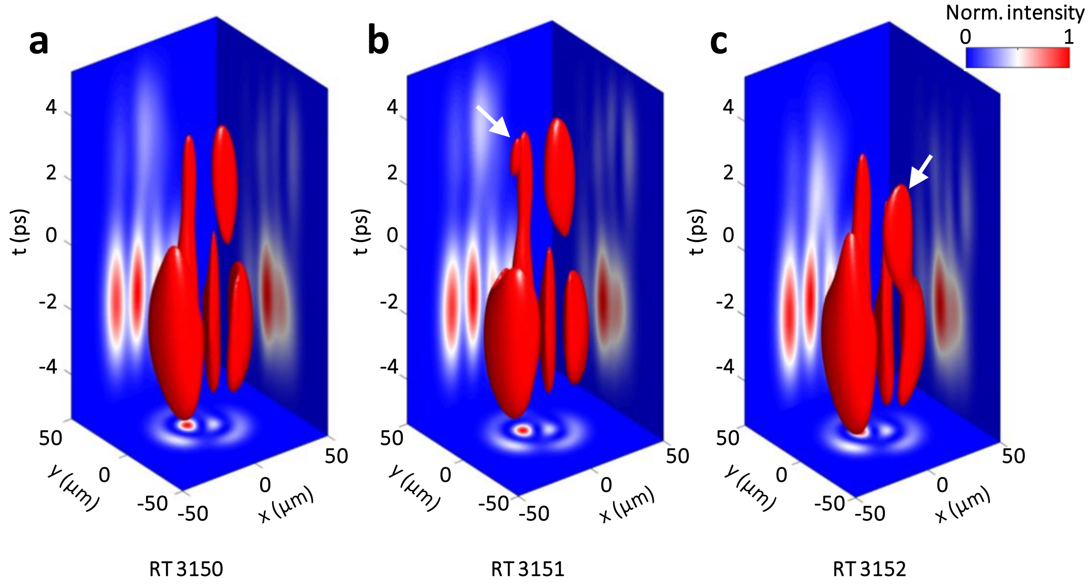

Supplementary Figure 21 | **Pulse-to-pulse spatiotemporal dynamics in the numerical simulation**. **a**. RT 3150. **b**. RT 3151. **c**. RT 3152. The arrows indicate the spatiotemporal variations. Here, the 3D isosurface plots are set to 10% of the peak field intensity.

Dual pulses with distinct group velocities can also be numerically achieved, as shown in **Supplementary Figure 22**, and the soliton collision is observed. Intriguingly, the

soliton collision leads to the explosion dynamics, after which subsequent pulsating decay occurs.

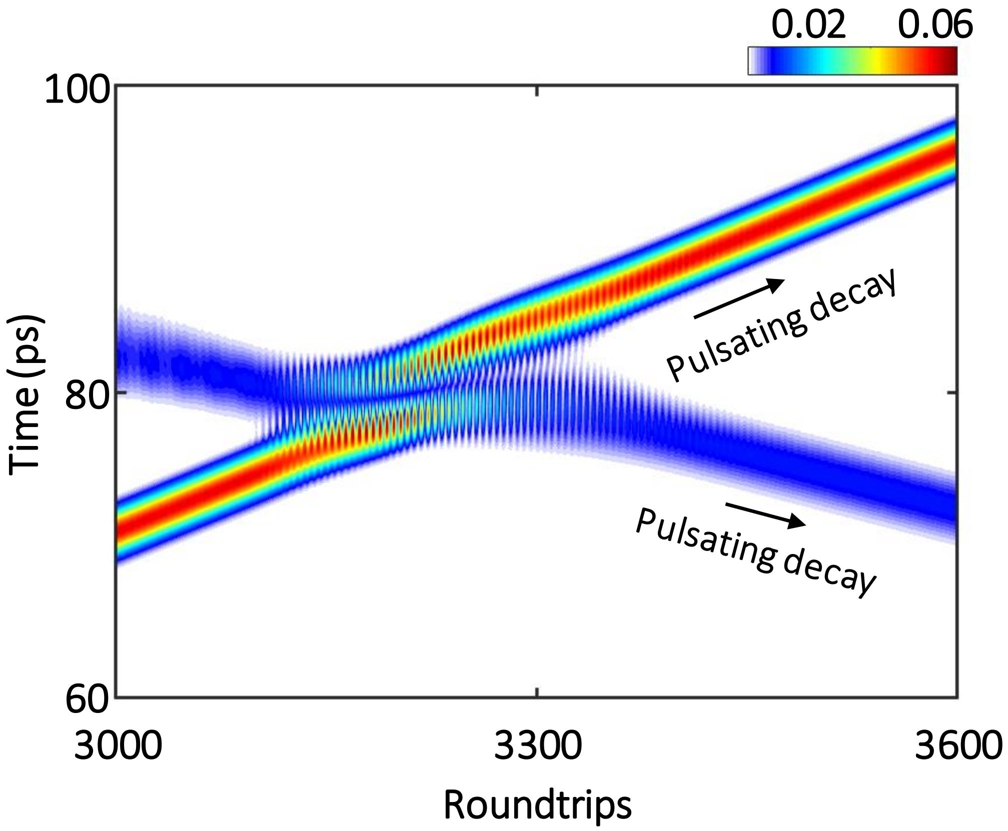

Supplementary Figure 22 | Explosion dynamics induced by 3D soliton collision.

Supplementary Note 10: Extended figures and tables

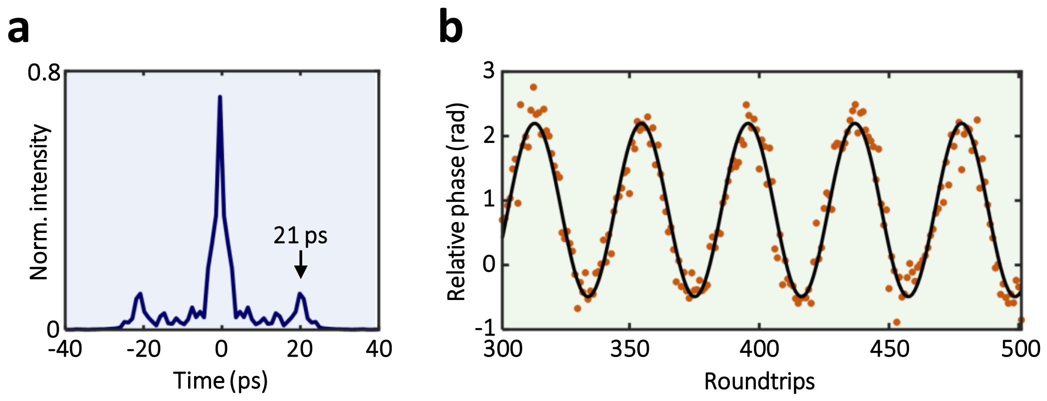

Supplementary Figure 23 | Characteristics of the snake-walking pulse P$_3$ in Fig. 6e. a. Typical FAC trace with a sidelobe at 21 ps. **b**. Spectral phase evolution of the snake-walking pulse. The dots represent the phases calculated from the measured spectral interferogram, while the solid curve is the sinusoidal fit.

Supplementary Table 3: Typical technologies for 3D lightwave characterization

Technology	If single shot	If burst mode	Temporal resolution	No. of continuous frames
Delay-scanning off-axis digital holography[6]	No	No	NA*	1**
TERMITES[9]	No	No	NA*	1**
SEA TADPOLE[10]	No	No	NA*	1**
STRIPED FISH[11]	Yes***	Yes	NA*	1
STS-CUP[12]	Yes	Yes	500 fs	60
CUST[13]	Yes	Yes	0.1–5 ps	60
T-CUP[14]	Yes	Yes	0.58 ps	350
ISIS CCD[15]	Yes	Yes	10 ns	16
FRAME[16]	Yes	Yes	200 fs	4
LIF-DH[17]	Yes	Yes	88 fs	7
SS-FTOP[18]	Yes	Yes	276 fs	4
MUST	Yes	No	12.5 ps and 47.8 ns****	>260,000*****

*Not provided in the paper.

**Multiple measurements are required for reconstructing the 3D structure.

***Single-shot measurement only for low repetition rate lasers.

****Here, the temporal resolution in the temporal domain is defined by the sampling rate of the real-time oscilloscope (80 GS/s), while the temporal resolution in the spectral domain is

defined by the repetition rate of the laser (20.9 MHz), i.e., 12.5 ps and 47.8 ns, respectively.
[*****]Here, the number of continuous frames is defined by the repetition rate of the laser (20.9 MHz) and memory depth of the real-time oscilloscope (1 Gpts/channel)

Supplementary Note 11: Scaling of the number of probes

To show the scalability of the sampling number, we have also conducted an experiment to demonstrate the speckly-diverse spectral-temporal dynamics at six spatial spots, as shown in **Supplementary Figure 24**. Based on the original design shown in **Supplementary Figure 1**, here another three single-mode probes (SMPs) are added. In brief, the laser signal from the laser cavity is first split into two branches by a 50:50 beam splitter (BS_2). In each branch, the laser signal is further split by two BSs with ratios of 30:70 (BS_3 and BS_5) and 50:50 (BS_4 and BS_6), respectively. The signals of three different SGs in the multimode laser beam are individually received by the SMPs in each branch. Then, the collected signals propagate through different ODLs, which are subsequently combined by a 3×2 OC for OTDM in each branch. The OTDM signal is split into two parts in each branch, one of which is directly detected by a high-speed PD (PD_1 in the top branch and PD_4 in the bottom branch). The other part is launched to the long SMF, which provides a large GVD (about -0.3 ns/nm) for real-time spectroscopy through photonic time stretch, in a counter-propagation scheme for the two branches. The optical loss is compensated by two Yb-doped fiber amplifiers (YDFAs). The time-stretched signal is detected by another two high-speed PDs (PD_2 and PD_3, respectively). The outputs of the PDs are finally recorded by a 4-channel real-time oscilloscope at a sampling rate of 80 GS/s.

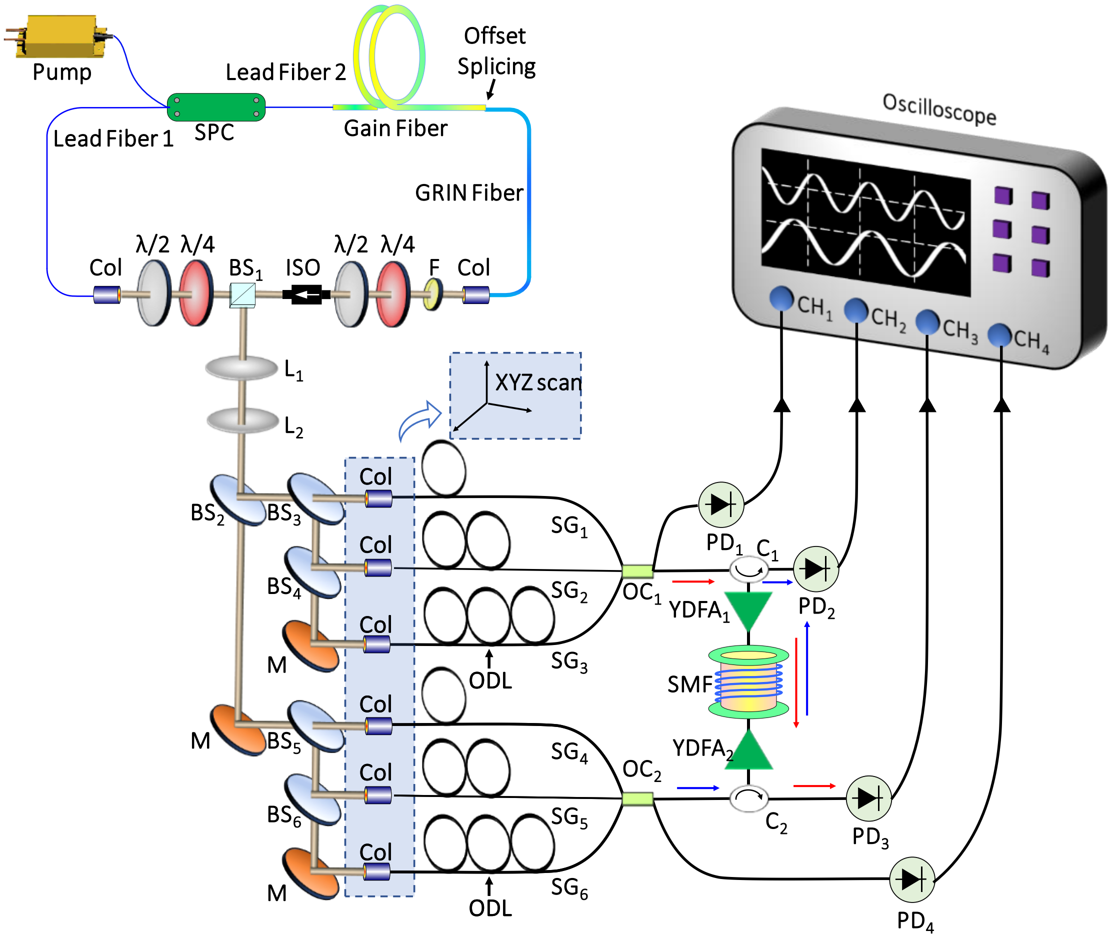

Supplementary Figure 24 | Configuration of the real-time MUST measurement system with six single-mode probes. BS: beam splitter. C: circulator. Col: collimator. F: filter. ISO: isolator. L: lens. M: mirror. OC: optical coupler. ODL: optical delay line. PD: photodiode. SMF: single-mode fiber. SG: speckle grain. SPC: signal-pump combiner. YDFA: Yb-doped fiber amplifier. λ/2: half-wave plate. λ/4: quarter-wave plate.

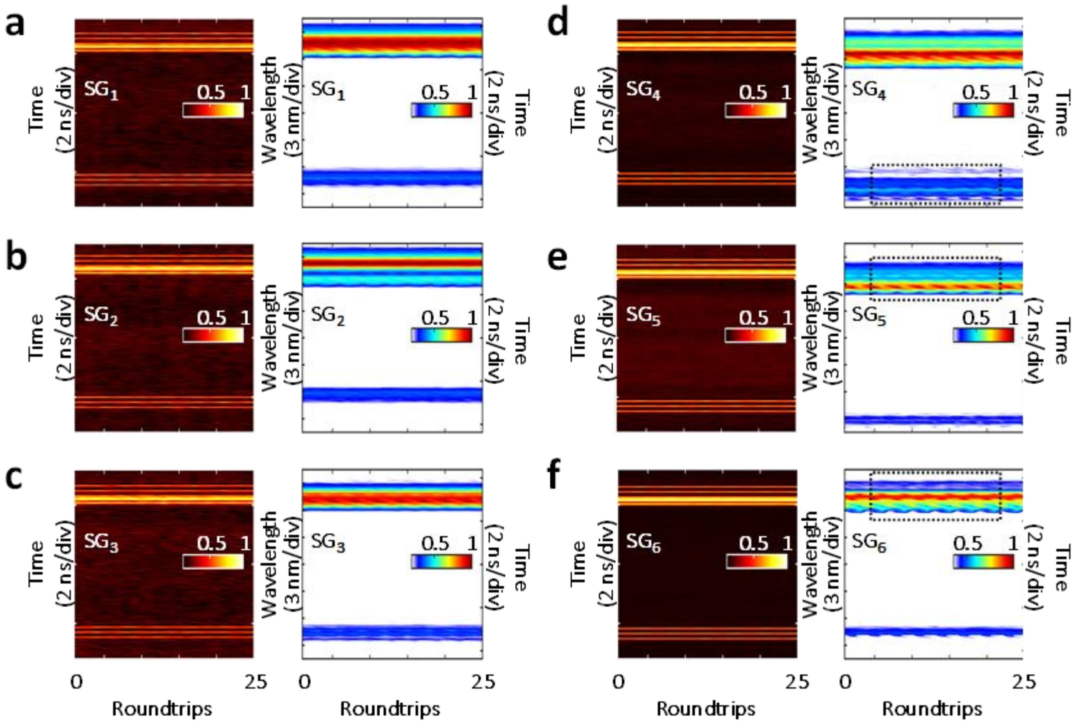

Supplementary Figure 25 | Temporal and spectral evolutions of the multipulse STML at six bright spots. **a**. Temporal (left) and spectral (right) evolutions of the multipulse cluster at speckle grain 1 (SG_1). **b**–**f**. Temporal and spectral evolutions of the multipulse cluster at SG_2–SG_6, respectively.

As shown in **Supplementary Figure 25**, the temporal evolutions of the six bright spots show similar multipulse landscapes in terms of pulse distribution and intensity modulation (mainly the three pulses at the bottom). However, the spectral evolutions of $SG_{4–6}$ are different from that of $SG_{1–3}$, as indicated by the dotted rectangles, where prominent spectral breathing similar to that of **Fig. 5** is visualized. Furthermore, the spectral evolutions of $SG_{4–6}$ exhibit different spectral dynamics. In other experiments, we observed the spectral interference with intensity modulation and phase drift using the same measurement system, as show in **Supplementary Figure 26a** and **b**, respectively.

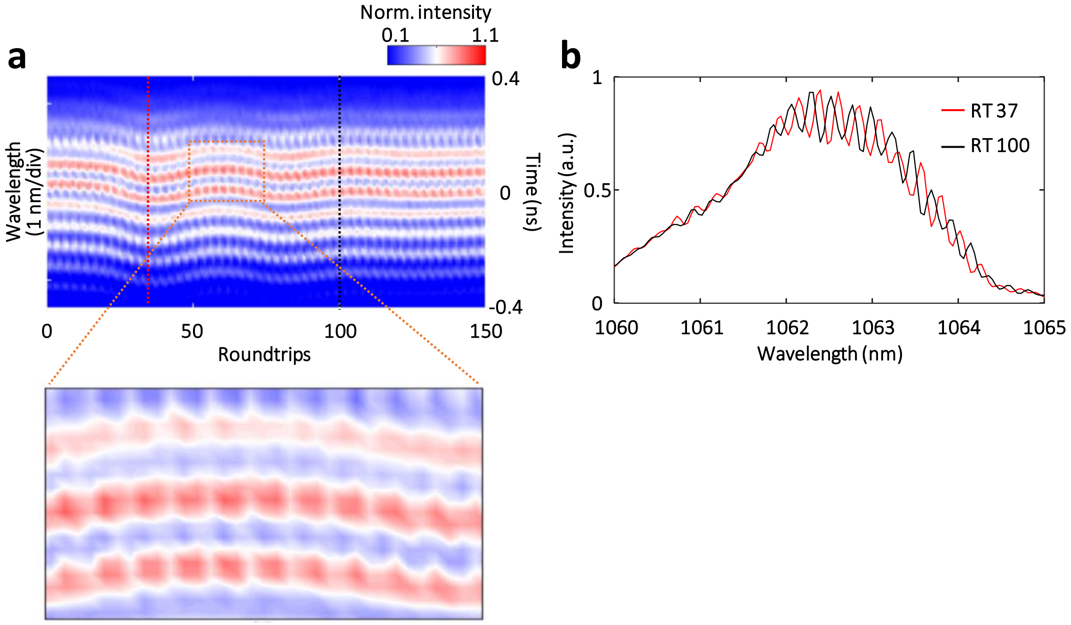

Supplementary Figure 26 | Spectral evolution of the multipulse STML. a. Spectral evolution of the multipulse STML at SG_3. Inset shows the close-up of the interference fringes. **b**. Single-shot optical spectra at RT 37 and 100, as indicated in **a**. Here, the results were captured by using the MUST measurement system with six probes.

Supplementary References

1. Mozjerin, I., Ruschin, S. & Hardy, A. A. Analysis of relaxation oscillations and gain switching of unidirectional erbium-doped waveguide ring lasers. *IEEE J. Quantum Elect.* **46**, 158–163 (2009).
2. Liu, X. & Cui, Y. Revealing the behavior of soliton buildup in a mode-locked laser. *Adv. Photonics* **1**, 016003 (2019).
3. Wang, Z. Q., Nithyanandan, K., Coillet, A., Tchofo-Dinda, P. & Grelu, P. Optical soliton molecular complexes in a passively mode-locked fibre laser. *Nat. Commun.* **10**, 830 (2019).
4. Krupa, K., Nithyanandan, K., Andral, U., Tchofo-Dinda, P. & Grelu, P. Real-time observation of internal motion within ultrafast dissipative optical soliton molecules. *Phys. Rev. Lett.* **118**, 243901 (2017).
5. Takeda, M., Ina, H. & Kobayashi, S. Fourier-transform method of fringe-pattern analysis for computer-based topography and interferometry. *J. Opt. Soc. A* **72**, 156–160 (1982).
6. Wright, L.G. et al. Mechanisms of spatiotemporal mode-locking. *Nat. Phys.* **16**, 565–570 (2020).
7. Poletti, F. & Horak, P. Description of ultrashort pulse propagation in multimode optical fibers. J. *Opt. Soc. Am. B* **25**, 1645–1654 (2008).
8. Shtyrina, O. V., Fedoruk, M. P., Kivshar, Y. S. & Turitsyn, S. K. Coexistence of collapse and stable spatiotemporal solitons in multimode fibers. *Phys. Rev. A* **97**, 013841 (2018).
9. Pariente, G., Gallet, V., Borot, A., Gobert, O. & Quéré, F. Space–time characterization of ultra-intense femtosecond laser beams. *Nat. Photon.* **10**, 547 (2016).
10. Bowlan, P., Gabolde, P. & Trebino, R. Directly measuring the spatio-temporal electric field of focusing ultrashort pulses. *Opt. Express* **15**, 10219–10230 (2007).

11. Gabolde, P. & Trebino, R. Single-shot measurement of the full spatio-temporal field of ultrashort pulses with multi-spectral digital holography. *Opt. Express* **14**, 11460–11467 (2006).
12. Jing, J. C., Wei, X., & Wang, L. V. Spatio-temporal-spectral imaging of non-repeatable dissipative soliton dynamics. *Nat. Commun.* **11**, 1–9 (2020).
13. Lu, Y., Wong, T. T., Chen, F. & Wang, L. Compressed ultrafast spectral-temporal photography. *Phys. Rev. Lett.* **122**, 193904 (2019).
14. Liang, J., Zhu, L., & Wang, L. V. Single-shot real-time femtosecond imaging of temporal focusing. *Light Sci. Appl.* **7**, 1–10 (2018).
15. Lazovsky, L., Cismas, D., Allan, G. & Given, D. CCD sensor and camera for 100 Mfps burst frame rate image capture. *Proc. SPIE* **5787**,184–190 (2005).
16. Ehn, A. et al. FRAME: femtosecond videography for atomic and molecular dynamics. *Light Sci. Appl.* **6**, e17045–e17045 (2017).
17. Kakue, T. et al. Digital light-in-flight recording by holography by use of a femtosecond pulsed laser. *IEEE J. Sel. Top. Quantum Electron.* **18**, 479–485 (2011).
18. Wang, X. et al. High-frame-rate observation of single femtosecond laser pulse propagation in fused silica using an echelon and optical polarigraphy technique. *Appl. Opt.* **53**, 8395–8399 (2014).
