# Supplementary material for: Real-time multispeckle spectral-temporal measurement unveils the complexity of spatiotemporal solitons
Source: Nat Commun. 2021 Jan 4;12:67. doi: 10.1038/s41467-020-20438-z (PMC7782776; doi:10.1038/s41467-020-20438-z)
Supplement: Supplementary file 3 — Description of Additional Supplementary Files [file 41467_2020_20438_MOESM3_ESM.pdf]

Description of Additional Supplementary Files

File Name: Supplementary Movie 1

Description: The evolution of the spatial mode profile during the transition from the CW to ML regime.
